# Inosine: biofunctions and the roles in human diseases

**DOI:** 10.3389/fendo.2025.1634814

**Published:** 2025-10-15

**Authors:** Fangwei Li, Jiawen Zhang, Jiayi Kang, Xuemei Tong, Ping Zhang, Yemin Zhu

**Affiliations:** Department of Biochemistry and Molecular Cell Biology, Shanghai Jiao Tong University School of Medicine, Shanghai, China

**Keywords:** inosine, metabolism, purinergic signaling, RNA editing, human diseases

## Abstract

Inosine, a basic component of purine nucleotide, is mainly seen only as a building block in nucleotide synthesis. It is also a versatile bioactive molecule with diverse biofunctions. These biofunctions are strongly related to human diseases or pathological conditions, such as cancer, obesity, inflammation, neurodegenerative diseases, and autoimmune diseases. In this article, we will discuss the roles and functions of inosine in a wide array of human diseases, targeting metabolism modulation, purinergic signaling, and RNA editing. We will also mention its great importance as a biomarker of human diseases. We believe that a more thorough understanding of inosine and its intricate roles in various human diseases could inspire future therapeutic methods or preventive modalities for these diseases.

## 1 Introduction

Inosine, comprising a purine (hypoxanthine) and a five-carbon sugar (ribose) (1), is widely known for its imperative role in nucleotide biosynthesis. The corresponding nucleotide, inosine 5′-monophosphate (IMP), functions as the biosynthetic precursor of adenosine 5′-monophosphate (AMP) and guanosine 5′-monophosphate (GMP) in the *de novo* pathway. Its base, hypoxanthine, is one of the main substrates in nucleotide salvage synthesis (2, 3).

**Figure 1:**
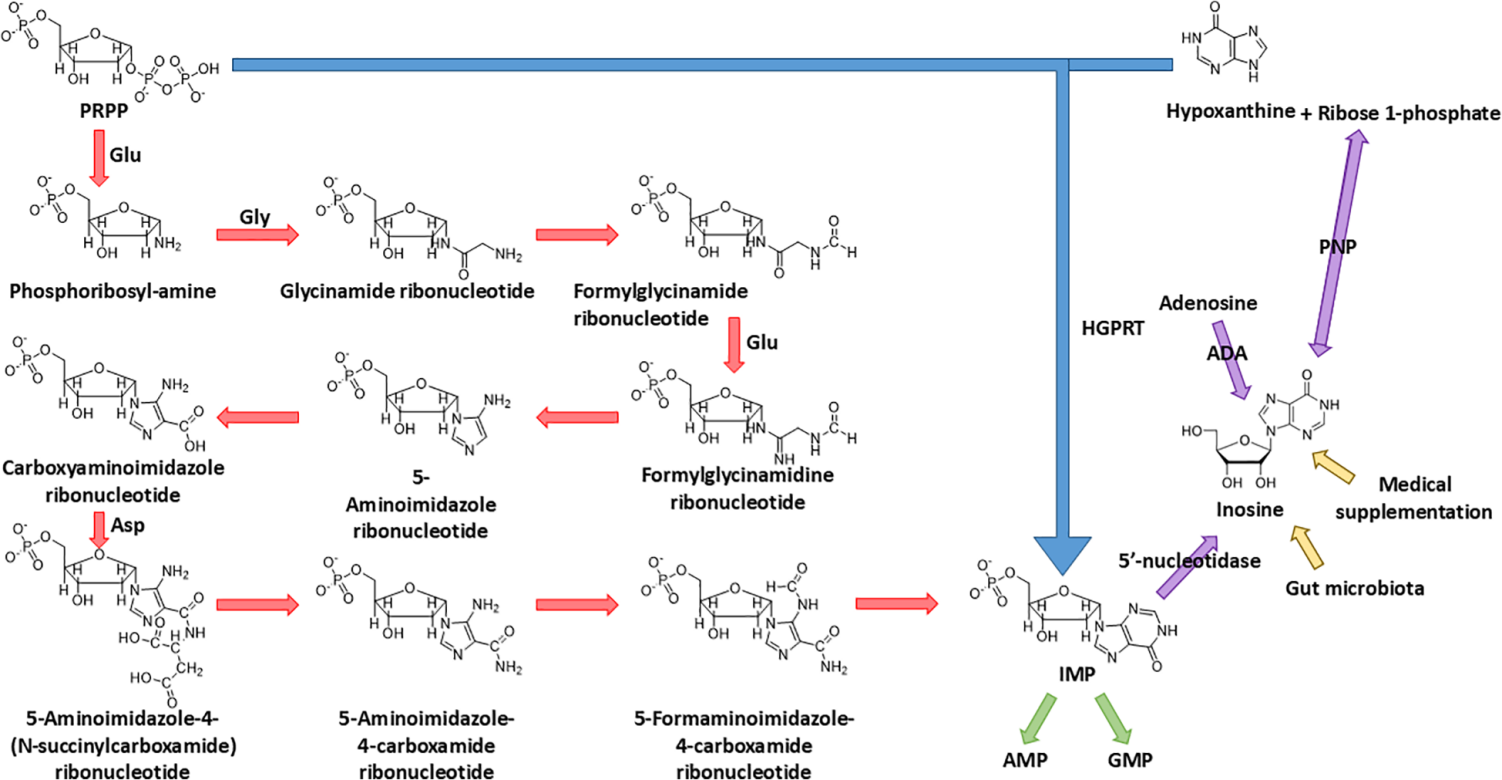
Biosynthesis of IMP and source of inosine. IMP is synthesized from both the *de novo* pathway (starting from PRPP) and the salvage pathway (salvaged from PRPP and hypoxanthine); the final product, IMP, of these two pathways could be dephosphorylated into inosine by 5'-nucleotidase. Other than dephosphorylation of IMP, the endogenous source of inosine also includes the deamination of adenosine and synthesis from ribose 1-phosphate and hypoxanthine. The exogenous source of inosine includes medical supplementation and excretion of gut microbiota.

To understand the metabolic origin of inosine, it is crucial to first examine IMP, a key purine nucleotide that serves as the precursor of inosine. IMP occupies a central role in both the *de novo* and salvage pathways of purine metabolism. In the *de novo* pathway, where nucleotides are built up from elemental constituents, IMP is meticulously constructed from 5-phosphoribosyl-1-pyrophosphate (PRPP). This scaffold facilitates the elaborate assembly of a range of amino acids through a sequence of reactions. Starting with glutamine PRPP amidotransferase catalyzing the donation of an amine group from glutamine to PRPP, the process advances through a four-step formation of the imidazole ring, followed by five subsequent reactions completing the pyrimidine structure. Upon its synthesis, IMP is capable of transforming into either AMP or GMP via amination. This transformation is under the careful control of glutamine PRPP amidotransferase, the enzyme that manipulates the equilibrium between feedback inhibition induced by purine nucleotides and the activation mediated by PRPP (2, 3) (**Figure 1**).

In the salvage pathway, where IMP undergoes a “restorative” process stemming from hypoxanthine, the enzyme hypoxanthine–guanine phosphoribosyl transferase (HGPRT) catalyzes the combination of PRPP and hypoxanthine, the base of inosine, to synthesize IMP in a simpler way (4) (**Figure 1**).

The source of inosine in the human body can be mainly divided into three parts: (1) The deamination of adenosine, catalyzed by adenosine deaminase (ADA), which is the major biological source of inosine generation (1). (2) The conversion from IMP. Enzyme 5′-nucleotidase catalyzes the dephosphorylation reaction of IMP to inosine. (3) The synthesis from hypoxanthine and ribose 1-phosphate (R1P) using purine nucleoside phosphorylase (PNP). Notably, under physiological conditions, inosine catabolism via phosphorolysis predominates over its synthesis. This occurs because (1) intracellular inorganic phosphate is far more abundant than nucleosides, and (2) hypoxanthine, the reaction product, is efficiently consumed by essentially irreversible downstream pathways—primarily salvaged by hypoxanthine–guanine phosphoribosyltransferase or oxidation by xanthine oxidase—which continually drive the reaction toward phosphorolysis (5–7) (**Figure 1**).

In addition, inosine can be incorporated into the human body exogenously. The exogenous inosine primarily comes from medical supplement and gut microbiota secretion (8). Notably, Mager et al. (9) verified this idea and isolated three strains of bacteria, *Bifidobacterium pseudolongum*, *Lactobacillus johnsonii*, and *Olsenella* sp., which significantly improve the immune checkpoint blockade treatment (**Figure 1**).

This review provides a comprehensive overview of inosine’s diverse biological functions. As a metabolic modulator, inosine serves as an alternative carbon source for CD8^+^ T cells under glucose-limiting conditions and influences energy metabolism in cancer cells. Beyond metabolism, inosine functions as a purinergic signaling molecule, engaging adenosine receptors to control obesity and regulate inflammation and neuroprotection. Subsequently, inosine contributes to post-transcriptional regulation as a nucleoside through adenosine-to-inosine RNA editing. Finally, inosine acts as a valuable biomarker for the diagnosis and prognosis of human diseases.

## 2 Inosine as a metabolic modulator

Emerging evidence reveals that inosine is a dynamic metabolic regulator with context-dependent functions in health and disease. Intracellularly, inosine serves as an alternative carbon source, fueling the tricarboxylic acid (TCA) cycle and sustaining ATP production under conditions of glucose deprivation or hypoxia (8). In cancer cells, inosine is capable of activating mechanistic target of rapamycin complex 1 (mTORC1), thereby promoting survival and growth during nutrient stress (10). Collectively, these findings position inosine as a versatile metabolite that bridges nucleotide homeostasis with broader metabolic networks, highlighting its potential as a therapeutic target in immunometabolic regulation and cancer metabolism.

### 2.1 Inosine as an alternative carbon source in immune cells

Bearing a five-carbon ribose moiety, inosine has the potential of being an excellent carbon source, evidenced by the ribose 5-phosphate (R5P) in the well-known pentose phosphate pathway (PPP). How inosine is incorporated into the PPP and the subsequent glycolysis pathway remains to be determined. This phenomenon is depicted by Wang et al. (8) using immune cells from both mouse and human. The phenomenon is based on the tumor microenvironment (TME), which showcases cancer cells’ intrusion within the host cell habitat, resulting in an assembly of diverse cell types (11). The TME is characterized by hypoxia and a dearth of nutrients due to the enhanced glucose consumption of cancer cells (12). This limitation in glucose availability can impair the function and survival of immune cells, which requires the rewiring of immune cells to adapt to this environment (13). One of them is the CD8^+^ T cell, which plays a crucial role in anti-tumor immune responses. Given the critical environment, CD8^+^ T cell relies on inosine as a compensatory energy substrate (8).

**Figure 2:**
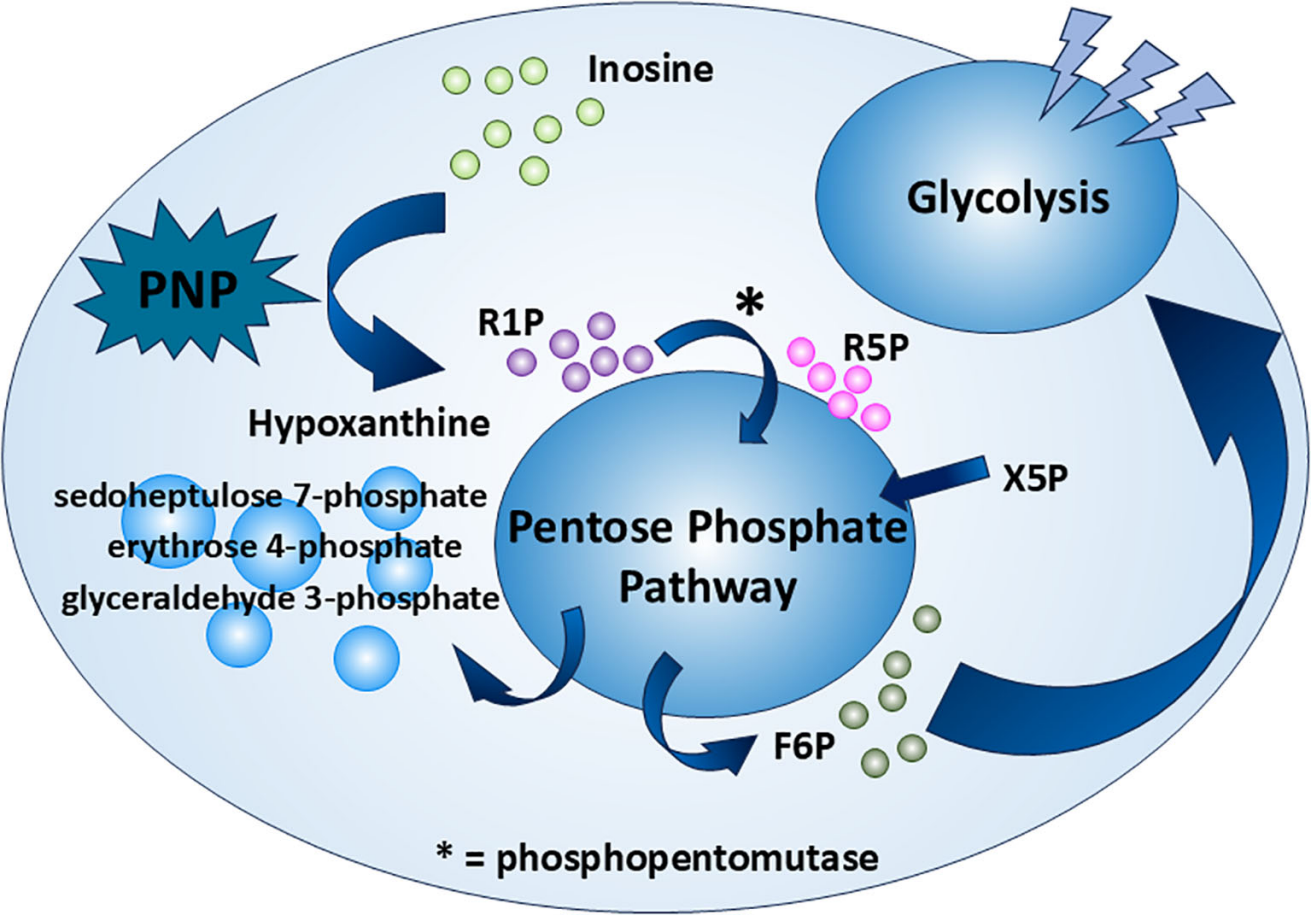
Inosine as an alternative carbon source. Inosine serves as a carbon source through the PPP and glycolysis under certain conditions. It is first broken down by PNP—a potential tumor therapeutic target—into R1P and hypoxanthine. R1P is then converted by phosphopentomutase into R5P, which enters the PPP and is ultimately converted to F6P. F6P then fuels glycolysis as a carbon source.

The inosine enters the CD8^+^ T cell though a protein transporter. However, both studies (8, 14) skip specifying the name of the transporter. For inosine to accomplish its role as a carbon source, the ribose moiety should be isolated before entering the PPP. The route of the inosine in the carbohydrate metabolism is tracked by a stable-isotope-based metabolomics approach. The ribose moiety of inosine is labeled with the ^13^C isotope ([1′,2′,3′,4′,5′-^13^C_5_]inosine) (8). It is shown that the ribose isolation step in CD8^+^ T cells is handled by the enzyme PNP, which converts inosine to hypoxanthine and R1P (**Figure 1**). As an interconvertible substrate with the R5P molecule, R1P is readily converted to R5P catalyzed by phosphopentomutase (7). R5P, an intermediate of PPP, undergoes the PPP along with xylulose 5-phosphate (Xu5P), providing an end product fructose 6-phosphate (F6P), which is one the important intermediates of the glycolysis pathway. Following the glycolysis pathway and subsequent TCA cycle, the ribose moiety of inosine achieves its role as a carbon source (**Figure 2**). In the same study (8), it is also reasoned that the PNP is an essential enzyme in the aforementioned process. Upon the use of a PNP inhibitor, forodesine (foro), a reduction in the bioenergetic activity, proliferation, viability, and tumor-killing activity is obvious in both mouse and human T effector cells cultured in inosine-containing medium. Furthermore, PNP is knocked down with PNP-targeted short-interfering RNA (siRNA), and the results follow the previous experiment. Both experiments suggest PNP as a crucial enzyme in inosine metabolism as a carbon source, which suggests a promising therapeutic strategy to manipulate T-cell metabolism and to enhance anti-tumor immune responses. There is another interesting point in this metabolic route. The hypoxanthine obtained from the breakdown of inosine by PNP, as mentioned before, is not only a nondescript by-product of the whole process as many may think. The uric acid derived from hypoxanthine has an anti-tumor effect by acting directly on CD8^+^ T cells (15, 16).

As inosine has this imperative role as an alternative carbon source in CD8^+^ T cells to fight against cancer, some microbiota that can produce inosine as metabolites show great enhancement in immune checkpoint blockade treatment (9, 17, 18).

### 2.2 Inosine as an energy modulator in cancer cells

In addition to its role in glycolysis (as a novel carbon source), inosine significantly influences mitochondrial respiratory capacity by integrating key metabolic pathways and signaling networks.

**Figure 3:**
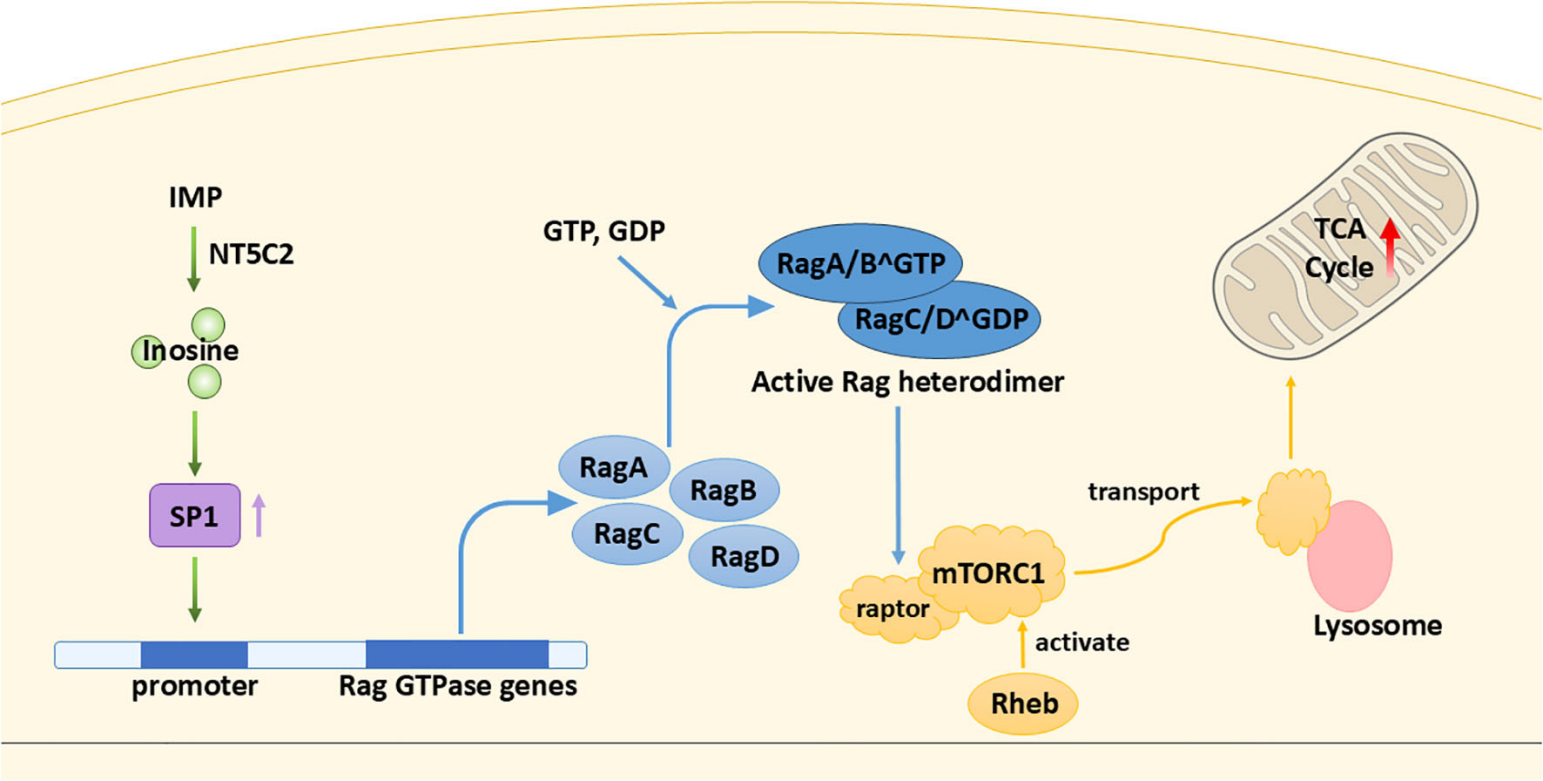
Inosine as an energy modulator. Inosine enhances the TCA cycle through a cascade of stimulatory interactions involving the transcription factor SP1, Rag GTPases, and mTORC1.

As mentioned earlier, the TME is characterized by hypoxia and nutrient scarcity, making alternative energy sources crucial. In such conditions, inosine helps buffer the energy deficit by modulating mitochondrial metabolism via the mTORC1 signaling pathway. In brief, mTORC1, one of the mTOR complexes, detects growth factors and abundance of nutrients and relays the signals to promote cell growth through stimulating biosynthesis (19). Li et al. (10) first find that inosine tends to accumulate in the tumor core region of certain tumors. Later experiments prove that the elevation of inosine promotes breast cancer cells’ (MDA-MB-231) survival in glucose- and glutamine-depleted conditions. The result is corroborated by a previous study, that inosine is capable of stimulating cancer cell proliferation (20). Different pathways are involved, which will be discussed shortly. In Li et al. (10), the source of inosine is endogenous, obtained from the breakdown of IMP by 5′-nucleotidase, cytosolic II (NT5C2) (21, 22). Cytosolic inosine enhances the level of the transcription factor specificity protein 1 (SP1) (the detailed mechanism has not been discovered yet); SP1 directly binds Ras-related GTP-binding proteins (Rag) GTPase gene promoters and enhances their transcription. There are four types of Rag GTPases: RagA, RagB, RagC, and RagD. The active Rag heterodimer (RagA/B^GTP^–RagC/D^GDP^) is able to bind to raptor, a subunit of mTORC1, and recruit it to the surface of the lysosome (23). At the lysosome, mTORC1 is activated by Rheb (a small GTPase) (24), leading to the upregulation of citrate synthase, aconitase 1, and other enzymes essential for the TCA cycle (10) (**Figure 3**). This enhancement of TCA cycle flux increases mitochondrial ATP production, supporting the energy demands of rapidly proliferating cancer cells, promoting the progression of cancer. However, in another study (20), it is suggested that inosine takes effect via binding to adenosine receptors A2B and A3 only (antagonists targeting the receptors A1 and A2A do not abolish the effect of inosine), possibly triggering the cyclic adenosine 5′-monophosphate–protein kinase A (cAMP–PKA) pathway.

Another question that may be asked is as follows: Given that glucose remains scarce despite the fact that TCA cycle enzymes are replete, what can the cancer cells “burn” for energy? In fact, less than 50% of the carbons in the acetyl coenzyme A (acetyl-CoA) pool are contributed by glucose (25). Cancer cells are able to utilize other carbon sources such as acetate, ketone bodies (25, 26), and inosine.

## 3 Inosine as a signaling molecule

Extracellular inosine is closely linked to a wide spectrum of human diseases. Once present in the extracellular space, inosine can modulate diverse cellular activities primarily by engaging adenosine receptors on the cell surface. Adenosine receptors are a family of G protein-coupled receptors (GPCRs) that mediate the physiological and pathological effects of adenosine and related nucleosides like inosine. There are four known subtypes: A1, A2A, A2B, and A3 (27). Through these GPCRs, inosine influences key signaling pathways that regulate energy expenditure, inflammatory response, and neuroprotection. In the following sections, we will further explore how receptor-specific interactions of inosine contribute to the development and progression of various human diseases.

### 3.1 The anti-obesity function of inosine

**Figure 4:**
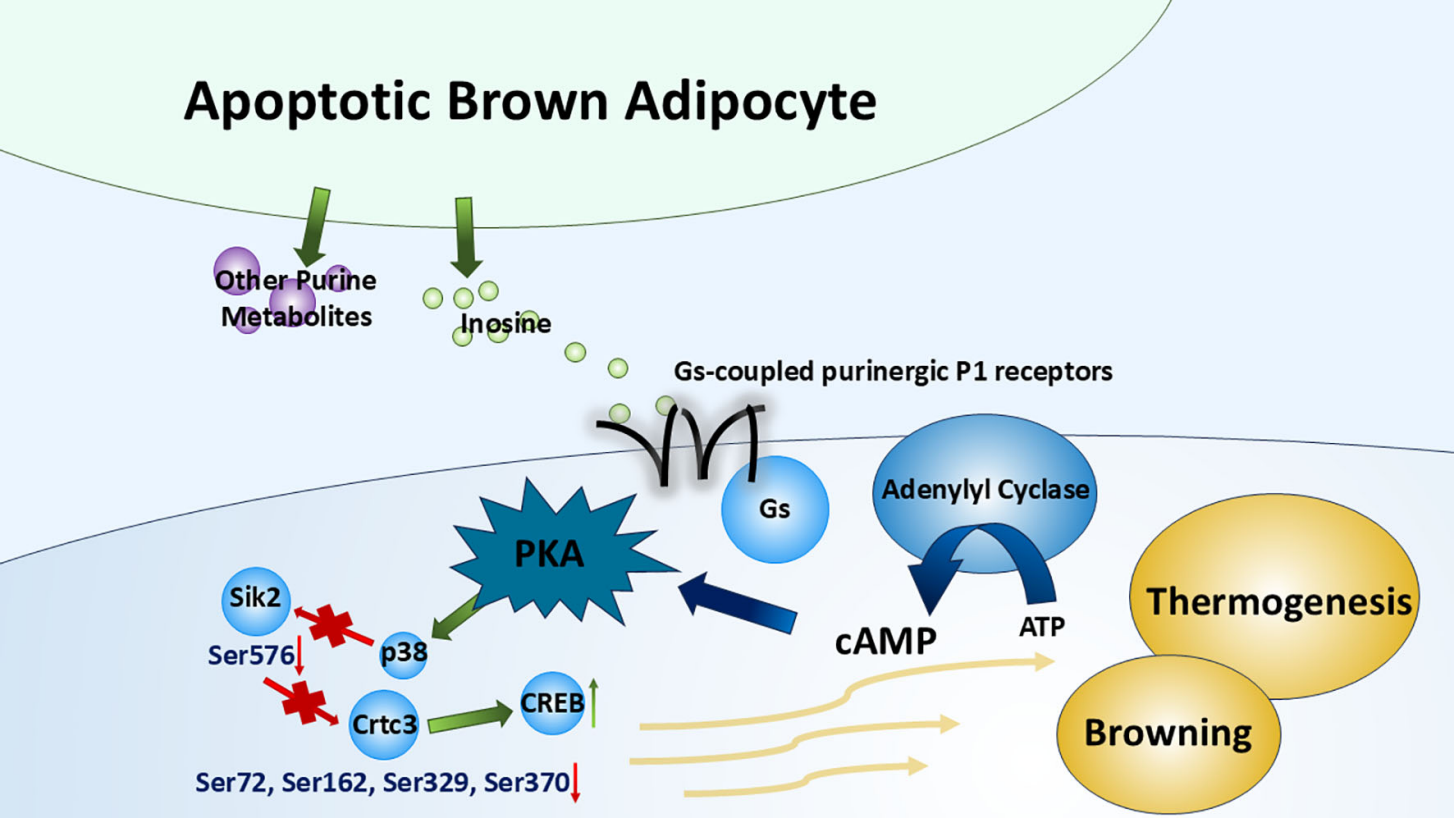
Inosine in thermogenesis of brown adipose tissue. By binding to G_s_-coupled purinergic P1 receptors (especially A2A and A2B receptors), inosine (released from apoptotic brown adipocytes along with several other purine metabolites) triggers the canonical cAMP–PKA cascade, which modulates multiple transcription factors or kinases. This cascade promotes the browning of WAT and the upregulation of BAT thermogenesis.

Rown adipose tissue (BAT) is a key site of non-shivering thermogenesis, which makes it an important therapeutic target for obesity and type 2 diabetes (28). Multiple factors can affect the browning/beiging of white adipose tissue (WAT) (into BAT) and the thermogenesis of BAT such as cold exposure (29), obesity (30), cancer cachexia (31), and diet (32). Niemann et al. (33) have identified inosine as a key regulator of BAT activation and thermogenesis in both humans and rodents. Apoptotic brown adipocytes release purine metabolites, including AMP, inosine, and hypoxanthine, among which only inosine has significant effects on the BAT, which is supported by another peer study (34). By binding to adenosine receptors, especially A2A and A2B, inosine increases intracellular cAMP levels, the main second messenger that boosts the differentiation process (WAT to BAT) and the thermogenesis within brown adipocytes. To be specific, PKA activated by cAMP further activates downstream targets such as p38 mitogen-activated protein kinase (p38 MAPK). It further reduces the phosphorylation of Salt-inducible kinase 2 (Sik2) (Ser576) and causes a subsequent decrease in phosphorylation of cAMP-regulated transcriptional coactivator 3 (Crtc3) (Ser72, Ser162, Ser329, and Ser370). This dephosphorylation has been proven to induce Crtc3 nuclear import and to instigate the association and activation of cAMP-responsive element binding protein 1 (Creb1) (35). These factors enhance the browning process of WAT and upregulate thermogenic gene, e.g., uncoupling protein 1 (UCP1) expression (36, 37) (**Figure 4**). Furthermore, inosine stimulates the mTORC1 and the MAPK extracellular signal-regulated kinase pathway, which are recognized for their roles in promoting brown adipocyte differentiation and the browning of WAT (33, 38). Given its role as a para- and autocrine signaling molecule driving energy expenditure in brown/beige adipocytes, inosine represents a promising target for anti-obesity interventions (33). Further efforts should focus on the efficacy and safety of this approach in preclinical models.

### 3.2 The anti-inflammatory effect of inosine

The relationship between adenosine receptors and inflammation has been well discovered. A large number of both *in vitro* and *in vivo* studies have shown the anti-inflammatory effects of adenosine receptors on multiple kinds of inflammatory cells and in different inflammatory diseases (39). However, inosine also has an effect on adenosine receptors, triggering similar anti-inflammation processes. Among the four subtypes of adenosine receptors, which receptors does inosine mainly bind to during the anti-inflammatory process remain to be determined. Haskó et al. (40) suggest that A1 and A2 receptors are mostly involved, while another study by Gomez and Sitkovsky (41) mentioned A2A and A3 receptors. The activation of these receptors suppresses pro-inflammatory cytokine production, mitigating inflammation-induced tissue damage and providing protection against endotoxin-induced shock (40, 41).

The study by Lima et al. (42) reveals the detailed mechanisms of the anti-inflammatory effect of inosine. In hypercholesterolemic rats (42), the expression of inducible nitric oxide synthase (iNOS) is high (43). The high expression of iNOS is associated with the stabilization of hypoxia-induced factor 1 (HIF-1) (43, 44), resulting in inflammation and oxidative stress. In addition, by synthesizing a large amount of nitric oxide (NO), iNOS can cause further tissue damage (43, 45). By binding particularly to the A2A and A3 receptors, inosine again promotes the cascade of cAMP–PKA. PKA phosphorylates the endothelial nitric oxide synthase (eNOS) at Ser1177. Upon phosphorylation, eNOS becomes active and produces NO in a moderate amount, which acts as a vasodilator, relaxing blood vessels and reducing blood pressure (42, 46). Additionally, NO signals the activation of the soluble guanylate cyclase (sGC), increasing cyclic guanosine 3′,5′-monophosphate (cGMP) levels, which then activates protein kinase G (PKG). PKG promotes vasodilation and protects against endothelial dysfunction (42). These findings are in accord with the effect of inosine on cAMP and PKA level in previous studies, contributing to its antiplatelet and anti-inflammatory abilities (47, 48).

Aside from the aforementioned PKA–eNOS–PKG pathway, another inosine-induced anti-inflammatory pathway [p38 MAPK/nuclear factor kappa-light-chain-enhancer of activated B cells (NF-κB)/vascular cell adhesion molecule-1 (VCAM-1)] has also been demonstrated by Lima et al. (42). p38 MAPK is a key stress-activated kinase that responds to inflammatory stimuli (49). In hypercholesterolemic rat models, activated p38 MAPK phosphorylates downstream targets, including transcription factors like NF-κB. NF-κB promotes the expression of adhesion molecules, e.g., VCAM-1 and a series of inflammatory genes, contributing to atherosclerosis progression (42, 49). After treatment with inosine, the level of the NF-κB inhibitor, inhibitor of nuclear factor kappa B, alpha (IκB-α), in the hypercholesteremic rat models shows an obvious increase. Accordingly, there is also a marked reduction in p38 MAPK, NF-κB, and VCAM-1 expression (42). Note that there is a contradiction here between this pathway and that mentioned in Niemann et al. (33), where p38 MAPK is activated by inosine. Our hypothesis is that either the hypercholesteremic state impacts it or other unexplained mechanisms are present; further studies are needed to elucidate this matter.

### 3.3 The neuroprotective role of inosine

Emerging evidence suggests that inosine enhances brain-derived neurotrophic factor (BDNF) signaling, which plays a vital role in neuronal survival, synaptic plasticity, and cognitive function in neurodegenerative diseases (50).

BDNF exerts its neuroprotective effects by activating the tropomyosin receptor kinase B (TrkB) receptor, a key mediator of long-term potentiation (LTP), which is essential for memory formation and synaptic transmission (51). It is indicated that the activation of adenosine receptors, particularly A1 and A2A, is necessary to initiate different BDNF-related functions (52). This activation ensures the maintenance of regular BDNF levels and the enhancement of BDNF-triggered enhancement of synaptic transmission within the hippocampus (53).

Many studies have shown the imperative role of inosine in enhancing the strength of BDNF upon binding to A1 and/or A2A receptors. In experimental models of Alzheimer’s disease (AD), induced by streptozotocin (STZ), the levels of TrkB receptor and BDNF mRNA expression in the hippocampus are drastically reduced. Notably, inosine administration significantly restored TrkB and BDNF levels (50). Blocking A1 or A2A receptors inhibits the inosine-induced increase in BDNF in brain (52, 54).

## 4 Inosine in RNA modification

Adenosine-to-inosine (A→I) RNA editing is one of the most widespread post-transcriptional modifications in cells. It is catalyzed by the adenosine deaminases acting on RNA (ADAR) family, which recognizes and binds double-stranded RNA (dsRNA) regions in precursor transcripts and hydrolytically deaminates specific adenosines to inosines (55, 56).

Chemically, ADARs remove the C6 amino group from adenosine, converting it into inosine (57). During translation and RNA processing, inosine is interpreted by the cell as guanosine, which can result in codon changes, alterations in splice sites, modulation of microRNA (miRNA) interactions, and shifts in RNA secondary structure and stability (58).

Editing events frequently occur in specific dsRNA regions such as the 3' untranslated regions (3'UTRs), transposable elements, or viral RNAs (59), and can be detected using high-throughput sequencing techniques such as RNA-seq (60).

Mammals express three ADAR family proteins: ADAR1 (also known as ADAR), ADAR2 (ADARB1), and ADAR3 (ADARB2). Among these, ADAR1 and ADAR2 exhibit enzymatic activity and are actively involved in A→I RNA editing. In contrast, ADAR3 has not shown any detectable editing activity on known RNA substrates and is generally considered to be enzymatically inactive (61, 62). This slight editing might not seem much; however, it could cause a significant aftermath when it goes awry. Mutations in ADAR1 have been discovered to be linked to a variety of diseases, while fewer diseases are related to mutations in ADAR2 (we still include a few in the following section). In the following section, we will review two rare diseases [dyschromatosis symmetrica hereditaria (DSH) and Aicardi–Goutières syndrome (AGS)], various types of cancers (63–65), and their relationship with ADAR family proteins.

### 4.1 A→I RNA editing defects: a trigger for DSH and AGS

DSH is a rare pigmentary genodermatosis, which is acquired by autosomal dominant inheritance with high penetrance. DSH typically presents with a mix of hyperpigmented and hypopigmented macules on the backs of the hands and feet, along with freckle-like lesions on the face. The rashes generally begin during infancy or early childhood (66).

AGS is a severe, genetically inherited neuroinflammatory disorder that mimics congenital viral infection. It typically presents in infancy with symptoms such as encephalopathy, intracranial calcifications, leukodystrophy, elevated interferon-α in the cerebrospinal fluid (CSF), and developmental delays (67).

Both diseases are found to be related to ADAR1. DSH has been found to be solely controlled by ADAR1 (68), while multiple genes have been found to be responsible for AGS, e.g., three prime repair exonuclease 1 (TREX1), ribonuclease h2 subunits a, b, and c (RNASEH2A–C), SAM domain and HD domain–containing protein 1 (SAMHD1), and interferon induced with helicase c domain 1 [IFIH1 or the protein it encodes, melanoma differentiation-associated protein 5 (MDA5)], including ADAR1 (69). The exact relationship between ADAR1 and DSH is still unclear. A few studies hypothesize that dysregulated RNA editing affects the expression of genes involved in melanocyte function, pigment distribution, or skin homeostasis (70–72), which leads to the pigmentary phenotype of DSH. AGS, on the other hand, is a result of an autoimmune response, caused by the accumulation of unedited self-RNA (73). Normally, A→I editing by ADAR1 stops the immune system from mistaking self-dsRNA as foreign by preventing its detection by sensors like MDA5, which triggers the mitochondrial antiviral signaling protein (MAVS) pathway and type I interferon production. Without proper editing, the body mistakes its own RNA for viral RNA, leading to constant interferon activation and inflammation, especially in the brain—a key feature of AGS (64, 74).

### 4.2 A→I RNA editing dysregulation: a double-edged sword in cancer

A → I RNA editing mediated by ADAR1 and ADAR2 is a vital post-transcriptional modification that reshapes both coding and noncoding RNAs in cancer. ADAR1-driven editing of coding transcripts—such as antizyme inhibitor 1 (AZIN1) (serine→glycine substitution)—enhances protein oncogenic potential across colorectal, endometrial, gastric, liver, and esophageal cancers (75–80). ADAR1 also impairs tumor suppressors [e.g., filamin B in triple-negative breast cancer, bladder cancer associated protein (BLCAP) in cervical cancer, and Nei-like DNA glycosylase 1 (NEIL1) in multiple myeloma], promoting proliferation, invasion, signal transducer and activator of transcription 3 (STAT3) activation, or genomic instability (81–83). In contrast, in contexts such as breast cancer, ADAR1-mediated editing of gamma-aminobutyric acid type A receptor subunit alpha 3 (GABRA3) attenuates protein kinase B (AKT) signaling and suppresses metastasis (84). The bimodal feature makes utilizing inosine as a novel therapeutic molecule more difficult, where more future studies are warranted.

Beyond coding RNAs, ADAR1 regulates noncoding RNAs—especially miRNAs and circular RNAs (circRNAs). In hepatocellular carcinoma (HCC), editing of miR-3144-3p by ADAR1 shifts its targeting away from tumor suppressors and toward oncogenes, and editing of pri-miR-26a or miR-200b in leukemia and thyroid cancer, respectively, disrupts miRNA maturation or epithelial–mesenchymal transition (EMT) suppression (85–88). ADAR1 also influences circRNA biogenesis by editing Alu-paired sequences, suppressing tumor-suppressive circRNA ARSP91 (circARSP91) in HCC, while certain circRNAs such as circRNA derived from the NEIL3 gene (circNEIL3) provide feedback to regulate ADAR1 expression (89–91).

ADAR2 generally functions as a tumor-suppressive RNA editor and is frequently downregulated in multiple cancers, with restoration of its editing activity reversing malignant phenotypes. In glioblastoma multiforme, low ADAR2 protein levels correlate with poorer overall survival, and suppression of ADAR2 enhances proliferation, migration, and anchorage-independent growth, and upregulates invasion-associated genes, e.g., a disintegrin and metalloproteinase domain-containing protein 12 (ADAM12), pentraxin 3, and EMT pathway genes (92). In esophageal squamous cell carcinoma, ADAR2 is reduced in tumors, and its overexpression induces apoptosis via editing and stabilizing insulin-like growth factor-binding protein 7 (IGFBP7), inhibiting Akt signaling. Knockdown of ADAR2 or IGFBP7 eliminates this pro-apoptotic effect (93). In the core-binding factor acute myeloid leukemia with t(8;21) or inv(16) fusions, transcriptional repression of ADAR2 by runt-related transcription factor 1 (RUNX1-ETO) reduces editing of key targets, coatomer protein complex subunit alpha (COPA), and component of oligomeric Golgi complex 3 (COG3). Loss of ADAR2 catalytic activity enhances leukemic clonogenicity (94).

## 5 Inosine as a biomarker of diseases

Inosine has emerged as a promising biomarker in a variety of human diseases due to its role in energy metabolism, oxidative stress response, and purinergic signaling. Recent studies have highlighted its diagnostic and prognostic potential across diverse pathological conditions, particularly when measured in plasma, urine, or feces. In cardiac ischemia, inosine concentrations in plasma rise sharply within minutes following the onset of myocardial injury, preceding traditional biomarkers such as troponin. This rapid elevation, detectable via high-performance liquid chromatography (HPLC) and chemiluminescence methods, offers the potential for ultra-early detection of ischemic events in emergency settings (95). In AD, fecal metabolomic analyses have identified significantly lower levels of inosine in patients compared to cognitively normal controls. A patented diagnostic model utilizing fecal inosine signal intensity has demonstrated high specificity and sensitivity, offering a novel, non-invasive approach for early AD detection (96). In the context of oncology, clinical metabolomics research has reported that plasma inosine levels correlate with lymph node metastasis, suggesting a role in cancer staging. Elevated inosine was associated with advanced nodal involvement, supporting its potential as a marker of disease dissemination in malignancies (97). In Kawasaki disease (KD), inosine has been identified as a component of biomarker panels capable of distinguishing patients with KD from healthy controls (98).

Collectively, these findings support the role of inosine as a dynamic and versatile biomarker across a range of diseases, with diagnostic applications spanning acute ischemia, neurodegeneration, cancer, and systemic inflammatory conditions.

## 6 Inosine as a novel therapeutic molecule: are we there yet?

According to the aforementioned novel functions of inosine, we believe that inosine will benefit the global population in terms of treating various diseases. As inosine supports T cells under glucose-restricted conditions, it is proved to be a promising molecule for treating cancer (8). As it promotes the “browning” of WAT, it could be utilized to alleviate obesity (33). It is also a versatile biomarker for multiple diseases, e.g., cardiac ischemia, AD, cancer, and KD. However, we are still not “there” yet. There are still a lot of mysteries waiting for us to discover. At the cellular level, the “preference” of inosine to different subtypes of adenosine receptors is still not well understood. Different studies, as mentioned before, have different results and opinions on this topic. We believe that further understanding of the ligand–receptor relationship can lead to the production of more selective medication regarding relevant diseases. In addition, different studies reveal discrepant relationships of inosine and p38 MAPK activation under different conditions. In one study (33), the p38 MAPK pathway is activated by inosine, while another study (42) tells a different story. The reason might be the physiological change in the model (hypercholesteremia) or other obscure mechanisms that still await discovery. In some diseases, such as cancer, inosine exhibits dual roles, functioning with both pro-tumorigenic and anti-tumorigenic effects; it could benefit the treatment by providing an alternative carbon source for CD8^+^ T cells (8) or escalating the progression of cancer through energy modulation (10). These two-sided features in its functions pose great obstacles to utilizing inosine in treating these diseases.

Future studies should also focus on the location, the dosage, and the delivery route to determine the effectiveness of inosine in treatment. Multiple randomized clinical trials show that orally administered inosine produces a rapid and sustained rise in serum (and sometimes CSF) urate, indicating considerable conversion of ingested inosine into downstream purine catabolites (xanthine → uric acid) (99, 100). This phenomenon can be attributed to the high abundance of PNP and xanthine oxidoreductase (XOR) in the human small intestine or liver, which facilitate the rapid conversion of luminal inosine into hypoxanthine and subsequently into xanthine/urate. Parenteral administration bypasses intestinal elimination; however, the liver remains a key site of inosine catabolism (101–104). Therefore, the question has been posed: Is inosine still “effective” if it is degraded to hypoxanthine/urate? It depends on the intended mechanism of action. If the therapeutic goal is to raise urate (e.g., for putative antioxidant/neuroprotective effects), metabolic conversion is actually the desired outcome: multiple clinical trials used oral inosine precisely to elevate systemic urate and demonstrated success in raising serum and CSF urate (Parkinson Study Group SURE-PD Investigators, 2014; 100). If the goal requires intact inosine acting directly on target cells (e.g., receptor-mediated signaling or intracellular inosine metabolism), parenteral delivery may improve the delivery of intact inosine—but this must be balanced against hepatic catabolism and safety. Human data and clinical outcomes with parenteral vs. oral inosine are scarce; more studies are needed in this field.

In this regard, while we maintain strong confidence in this novel and promising therapeutic molecule, much work remains to reveal the still hidden secret of inosine, and we hope that its benefits will extend to a broader range of human diseases.

## 7 Summary

In this review, we discuss some of the latest discoveries regarding inosine, including its roles in metabolism regulation, purinergic signaling, and post-transcriptional regulation (A→I RNA editing) and as a biomarker of multiple diseases. We demonstrate that inosine is associated with a huge array of human diseases or pathological conditions, including cancer, obesity, inflammation, neurodegenerative diseases, autoimmune diseases, and cardiovascular diseases. Our paper highlights the promising future of inosine as a novel therapeutic molecule. However, since certain mechanisms remain unclear and further clinical trials are necessary, it may still take some time before inosine becomes available in clinical practice. We hope that there will be more studies in the future that determine the detailed mechanisms of, e.g., the purinergic pathway and the pathway of how A→I RNA editing influences diseases, or that focus on clinical trials of inosine-related treatment.
